# Virotherapy of Canine Tumors with Oncolytic Vaccinia Virus GLV-1h109 Expressing an Anti-VEGF Single-Chain Antibody

**DOI:** 10.1371/journal.pone.0047472

**Published:** 2012-10-16

**Authors:** Sandeep S. Patil, Ivaylo Gentschev, Marion Adelfinger, Ulrike Donat, Michael Hess, Stephanie Weibel, Ingo Nolte, Alexa Frentzen, Aladar A. Szalay

**Affiliations:** 1 Department of Biochemistry, University of Wuerzburg, Wuerzburg, Germany; 2 Genelux Corporation, San Diego Science Center, San Diego, California, United States of America; 3 Small Animal Clinic, University of Veterinary Medicine, Hannover, Germany; 4 Rudolf Virchow Center for Experimental Biomedicine, University of Wuerzburg, Wuerzburg, Germany; 5 Institute for Molecular Infection Biology, University of Wuerzburg, Wuerzburg, Germany; 6 Department of Radiation Oncology, Moores Cancer Center, University of California San Diego, La Jolla, California, United States of America; Meharry Medical College, United States of America

## Abstract

Virotherapy using oncolytic vaccinia virus (VACV) strains is one promising new strategy for cancer therapy. We have previously reported that oncolytic vaccinia virus strains expressing an anti-VEGF (Vascular Endothelial Growth Factor) single-chain antibody (scAb) GLAF-1 exhibited significant therapeutic efficacy for treatment of human tumor xenografts. Here, we describe the use of oncolytic vaccinia virus GLV-1h109 encoding GLAF-1 for canine cancer therapy. In this study we analyzed the virus-mediated delivery and production of scAb GLAF-1 and the oncolytic and immunological effects of the GLV-1h109 vaccinia virus strain against canine soft tissue sarcoma and canine prostate carcinoma in xenograft models. Cell culture data demonstrated that the GLV-1h109 virus efficiently infect, replicate in and destroy both tested canine cancer cell lines. In addition, successful expression of GLAF-1 was demonstrated in virus-infected canine cancer cells and the antibody specifically recognized canine VEGF. In two different xenograft models, the systemic administration of the GLV-1h109 virus was found to be safe and led to anti-tumor and immunological effects resulting in the significant reduction of tumor growth in comparison to untreated control mice. Furthermore, tumor-specific virus infection led to a continued production of functional scAb GLAF-1, resulting in inhibition of angiogenesis. Overall, the GLV-1h109-mediated cancer therapy and production of immunotherapeutic anti-VEGF scAb may open the way for combination therapy concept i.e. vaccinia virus mediated oncolysis and intratumoral production of therapeutic drugs in canine cancer patients.

## Introduction

Cancer is the leading cause of disease-related death in dogs worldwide ([1], National Canine Cancer Foundation). Incidence of cancer ranges from 1 to 2% in the canine population and is currently the leading cause of deaths in dogs older than 10 years [1]–[2]. The major treatment options available for canine cancers include surgery, radiation therapy, chemotherapy, hyperthermia and photodynamic therapy. Despite progress in the diagnosis and treatment of advanced canine cancer, overall patient treatment outcome has not substantially improved in the past. Therefore, the development of new therapies for advanced canine cancer is a high priority. One of the most promising novel cancer therapies is oncolytic virotherapy. This method is based on the capacity of oncolytic viruses (OVs) to preferentially infect and lyse cancer cells without causing excessive damage to surrounding normal tissues. Several oncolytic viruses including various human and canine adenoviruses, canine distemper virus (CDV) and vaccinia virus strains have been successfully tested for canine cancer therapy in preclinical settings (for review see [3]).

**Figure 1:**
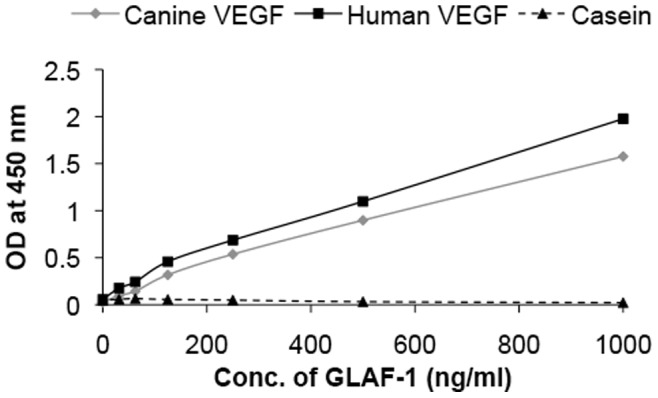
Interaction of purified GLAF-1 antibody with human and canine VEGF. Affinity and cross reactivity of GLAF-1 was demonstrated by ELISA. Equal concentrations of human or canine VEGF (100 ng/well) were coated on ELISA plates. Six two-fold dilutions of purified GLAF-1 protein ranging from 1000 ng/ml to 31.3 ng/ml were incubated with both human and canine VEGF. Plates pre-coated with 1% (w/v) casein (Pierce, 37528) were used as negative control. For further ELISA experimental conditions see material and methods (GLAF-1 ELISA). ODs obtained for various conc. of GLAF-1 against both human and canine VEGF were plotted. ELISA was repeated (n = 3) in an independent experiment.

In this study, we analyzed the therapeutic potential of the oncolytic vaccinia virus GLV-1h109 strain in two different xenograft models based on canine soft tissue sarcoma STSA-1 cells [4] and canine prostate carcinoma DT08/40 cells [5]. GLV-1h109 virus was derived from the oncolytic vaccinia virus GLV-1h68 [6] by replacing *lacZ* gene (beta-galactosidase) with GLAF-1 protein encoding gene at *J2R* locus [7]. The *glaf-1* gene encodes the single chain anti-VEGF antibody. GLAF-1 protein, comprises of an Igê light chain leader sequence [8], the V_H_ chain sequence of the G6–31 antibody [9], a (G_4_S)_3_ linker sequence, the V_L_ chain sequence of the G6–31 antibody [9], and a C-terminal DDDDK sequence [7]. The G6–31 antibody binds both murine (mu) and human (hu) vascular endothelial growth factor (VEGF) with high affinity [9]. The GLAF-1 antibody encoded by VACV strain GLV-h109 is expressed under the control of the vaccinia virus synthetic late (SL) promoter and also recognizes specifically mu and huVEGF [7]. However, cross reactivity of GLAF-1 with VEGF protein from other species was not known. VEGF or VEGF-A is a potent regulator of angiogenesis and therefore several anti-VEGF strategies have been developed for the treatment of human and canine tumors [10] [11], [12]. One of the best characterized strategies is the VEGF blockade using the humanized anti-VEGF monoclonal antibody (mAb) bevacizumab (avastin). However, despite very promising preclinical results, bevacizumab has not been shown to provide a benefit in patients with breast cancer (http://www.fda.gov/NewsEvents/Newsroom/PressAnnouncements/ucm279485.htm) or when used in combination with chemotherapy for the treatment of colorectal cancer and non-small-cell carcinoma in humans [13]. The molecular and cellular events underlying resistance to anti-VEGF antibody-based therapy are not completely understood [14]. However, the lack of efficacy of bevacizumab after systemic treatment in patients may be at least attributable to the poor penetration of this antibody into the tumor tissue and metastases. Therefore, new methods or vectors allowing more specific delivery of the anti-VEGF antibodies into the tumor tissue are urgently necessary. We have already shown that the recombinant Vaccinia virus strains (VACV) expressing the GLAF-1 antibody exhibited enhanced tumor inhibition and therapeutic potency, which was comparable to the results seen in combination therapy with separately injected bevacizumab and the parental virus GLV-1h68 [7]. Therefore, the GLV-1h109 virus dependent, intratumoral expression of GLAF-1 may become a new method allowing more optimal continuous delivery of this antibody into tumor tissues in dogs.

Here, we report the virus-mediated oncolytic and immunological effects upon colonization of GLV-1h109 and the constitutive intratumoral GLAF-1 production in STSA-1- and DT08/40 tumor bearing xenograft mice monitored by fluorescence imaging, Western Blot analysis, immunohistochemistry, flow cytometry and ELISA.

## Materials and Methods

### Ethics Statement

All animal experiments were carried out in accordance with protocols approved by the Institutional Animal Care and Use Committee (IACUC) of Explora Biolabs (San Diego, CA, USA; protocol number: EB11-025) and/or the government of Unterfranken, Germany (permit number: 55.2-2531.01-17/08).

**Figure 2:**
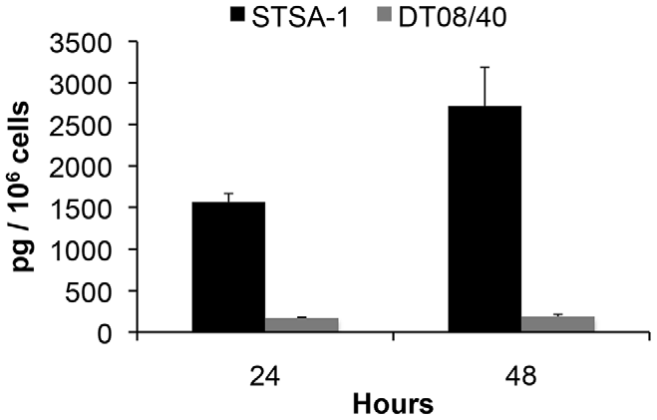
VEGF expression in STSA-1 and DT08/40 canine cancer cells under cell culture conditions. STSA-1 and DT08/40 cells in culture conditions were washed with PBS and cultured in fresh medium with 2% FCS. Culture supernatants were harvested at 24 and 48 h. VEGF levels in supernatants were determined by canine VEGF Quantikine ELISA kit (R&D Systems, Minneapolis, MN, USA). Each value represents the mean (n = 3) +/- standard deviations (SD).

### Cell Culture

African green monkey kidney fibroblasts (CV-1) were obtained from the American Type Culture Collection (ATCC). STSA-1 cells were derived from a canine patient with a grade II soft tissue sarcoma [4]. DT08/40 is a canine prostate carcinoma cell line [5].

CV-1 and DT08/40 cells were cultured in DMEM supplemented with antibiotic-solution (100 U/ml penicillin G, 100 units/ml streptomycin) and 10% fetal bovine serum (FBS; Invitrogen GmbH, Karlsruhe, Germany) for CV-1 and 20% FBS for DT08/40. STSA-1 cells were cultured in minimum essential medium with Earle’s salts supplemented with 2 mM glutamine, 50 U/mL penicillin G, 50 µg/mL streptomycin, 1 mM sodium pyruvate, 0.1 mM nonessential amino acids (MEM-C), and 10% FBS. All cell lines were cultured at 37°C in a 5% CO_2_ humidified incubator.

**Figure 3:**
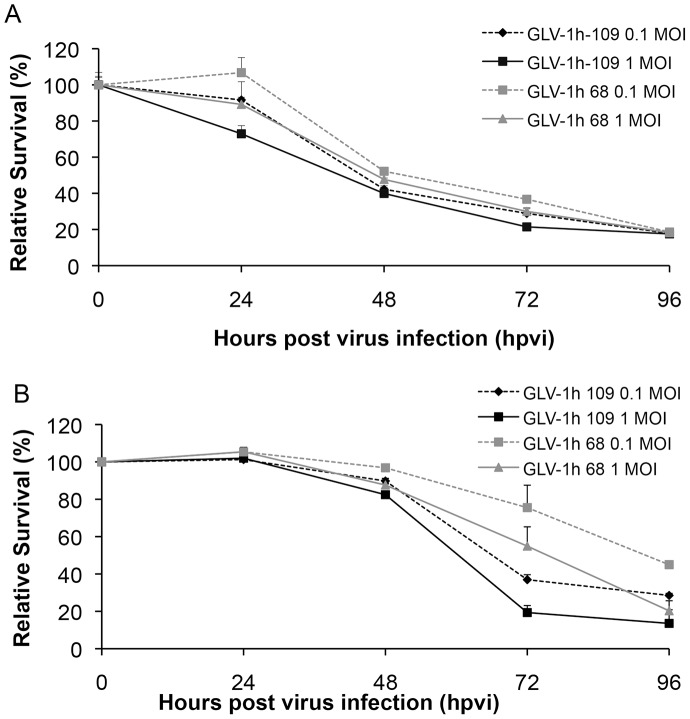
Viability of (A) canine soft tissue sarcoma (STSA-1) and (B) prostate carcinoma (DT08/40) cells after GLV-1h109 or GLV-1h68 infection. Viable cells after infections with two viruses at MOIs of 0.1 and 1.0 were detected using a XTT assay (Cell Proliferation Kit II, Roche Diagnostics, Mannheim, Germany) (Sigma, Taufkirchen, Germany). Mean values (n = 3) and standard deviations are shown as percentages of respective controls. The data represent three independent experiments. There were no significant differences between groups at 72 and 96 hpvi (P>0.05).

### VEGF ELISA

For the quantitative determination of canine VEGF concentrations in STSA-1 and DT08/40 cell culture supernatants; 5×10^6^ cells were cultured under the conditions of MEM and DMEM respectively, containing 10% FBS. Cell culture supernatants were collected at 24 and 48 h and stored at −20°C. Concentrations of VEGF were determined by canine VEGF Quantikine ELISA kit (R&D Systems, Minneapolis, MN, USA) according to the manufacturer’s protocol.

**Figure 4:**
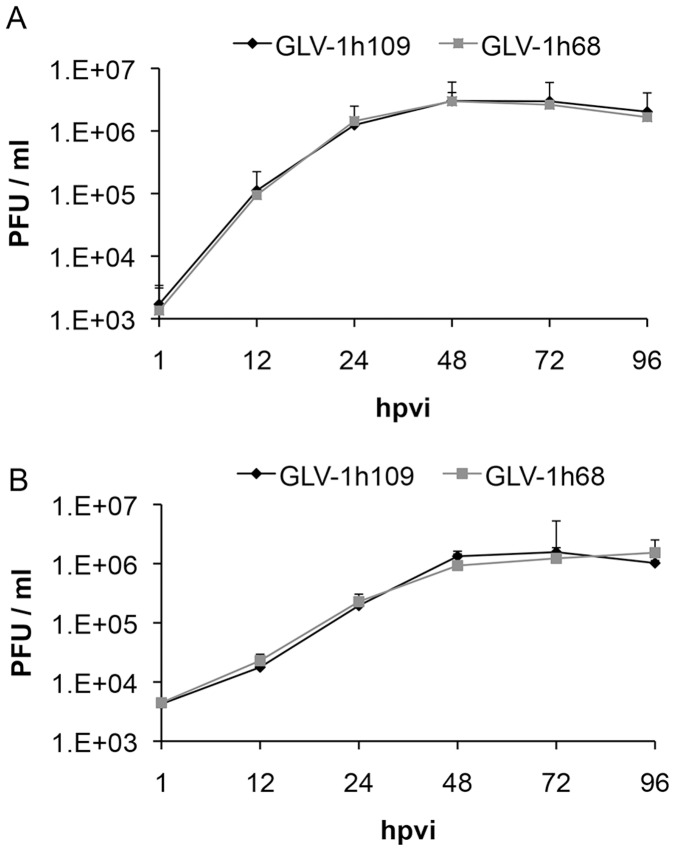
Comparison of the replication capacity of the vaccinia virus strains GLV-1h109 and GLV-1h68 in canine cancer cells. For the viral replication assay, STSA-1 (**A**) or DT08/40 (**B**) cells grown in 24-well plates were infected with either GLV-1h109 or GLV-1h68 at a MOI of 0.1. Cells and supernatants were collected for the determination of virus titers at various time points. Viral titers were determined as pfu per well in triplicates by standard plaque assay in CV-1 cell monolayers. Averages plus standard deviation are plotted. The data represent three independent experiments.

### Virus Strains

GLV-1h68 is an oncolytic vaccinia virus strain designed to locate, enter, colonize and destroy cancer cells without harming healthy tissues or organs [6]. GLV-1h109 is a GLV-1h68-derivative expressing an anti-VEGF single-chain antibody GLAF-1 [7].

**Figure 5:**
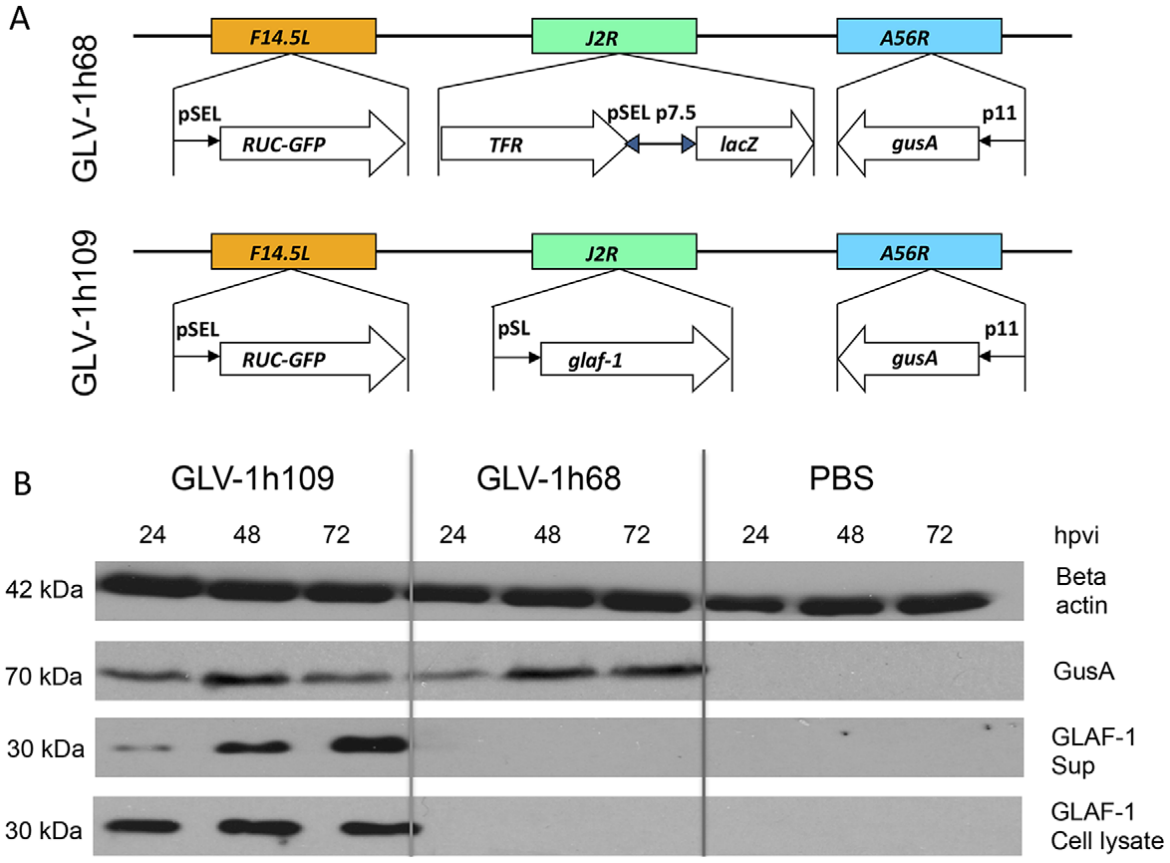
Expression of virus mediated proteins GLAF-1 and GusA in canine soft tissue sarcoma STSA-1 cells. (**A**) Schematic representation of GLV-1h68 and GLV-1h109 according to Frentzen et al [7]. abberations: p11, VACV p11 late promoter; pSEL, VACV SEL promoter; pSE, VACV SE promoter; pSL, VACV SL promoter; p7.5, VACV 7.5 K early/late promoter. (**B**) Western blot analysis of STSA-1 cells infected with either GLV-1h109, GLV1h 68 virus at an MOI of 1 or PBS. Protein fractions from cell lysate and culture supernatant were isolated at different time points and separated by SDS/PAGE. Western blot analysis was performed as described in material and methods.

### Cell Viability Assay

1×10^4^ cells/well were seeded in 96-well plates (Nunc, Wiesbaden, Germany). After 24 h in culture, cells were infected with vaccinia virus strains using multiplicities of infection (MOI) of 0.1 and 1.0. The cells were incubated at 37°C for 1 h, then the infection medium was removed and subsequently the cells were incubated in fresh growth medium.

**Figure 6:**
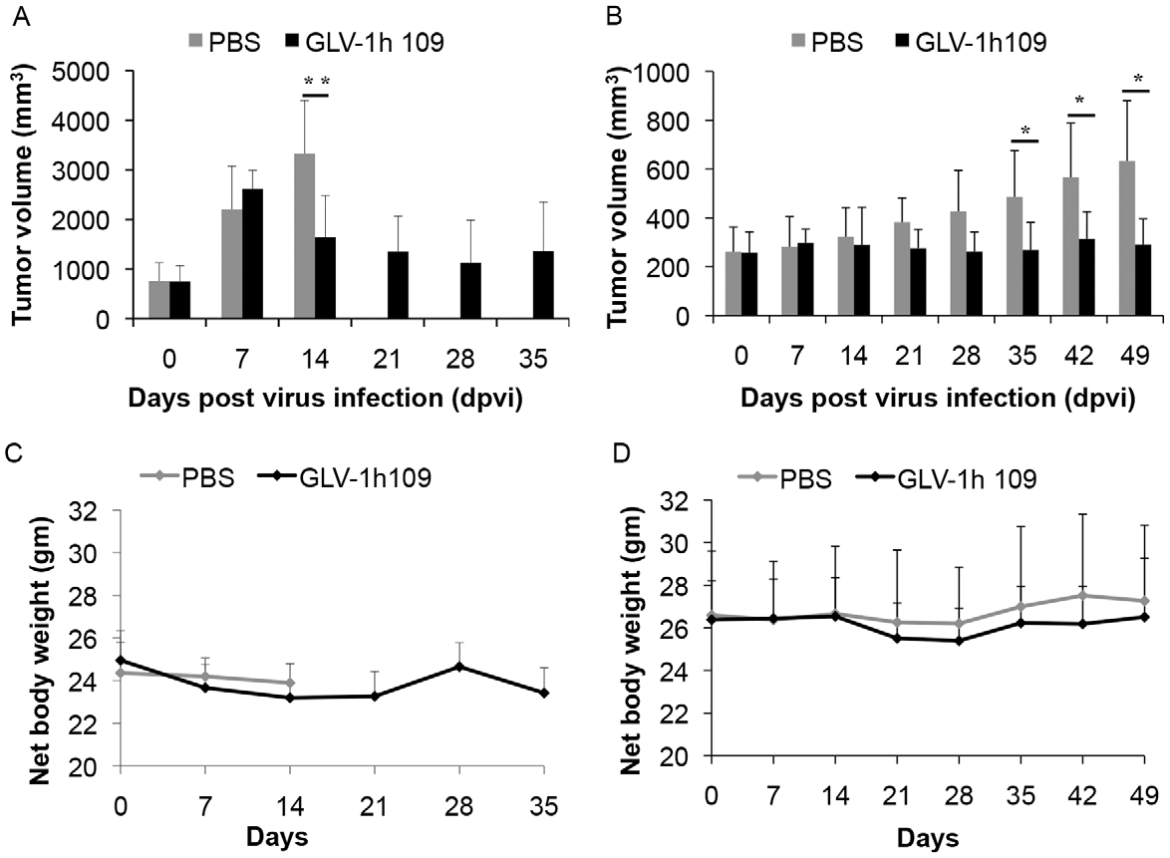
Effects of systemic GLV-1h109 virus injection on tumor growth (A, B) and the body weights (C, D) of STSA-1 or DT08/40 xenografted mice. Two groups each of (**A, C**) STSA-1 tumor-bearing nude mice (n = 6) and (**B, D**) DT08/40 tumor bearing mice (n = 6) were either treated with a single dose of 5×10^6^ pfu GLV-1h109 or with PBS (mock control) intravenously (i.v.) into lateral tail vein. The statistical significance was confirmed by Student’s t-test where * and ** indicate P<0.05 and 0.01 respectively.

The amount of viable cells after infection was measured using 2,3-bis[2-methoxy-4-nitro-5-sulfophenyl]-2*H*-tetrazolium-5-carboxanilide inner salt (XTT) assay (Cell Proliferation Kit II, Roche Diagnostics, Mannheim, Germany), according to the manufacturer’s protocol at 24, 48,72 or 96 h after infection. Quantification of cell viability was performed in an ELISA plate reader (Tecan Sunrise, Tecan Trading AG, Austria) at 490 nm with a reference wavelength of 690 nm. The relative number of viable cells was expressed as percent cell viability. Uninfected cells were used as reference and were considered as 100% viable.

**Table 1:** Biodistribution of GLV-1h109 in virus-treated DT08/40-or STSA-1 xenografts at 49 or 35 days post virus injection (dpvi).

PFU/per gram (g) oforgan or tumor tissue	DT08/40 xenograft			STSA-1 xenograft		
Mouse No/dpvi	424/49 dpvi	429/49 dpvi	433/49 dpvi	329/35 dpvi	343/35 dpvi	335/35 dpvi
**Tumor**	2.67E+02	4.53E+03	1.28E+04	1.86E+07	4.95E+07	2.70E+07
**Liver**	n.d	n.d	n.d	5.38E+01	1.19E+02	4.87E+01
**Lung**	n.d	n.d	n.d	2.65E+02	9.60E+01	1.05E+02
**Heart**	n.d	n.d	n.d	n.d	n.d	n.d
**Kidney**	n.d	n.d	n.d	5.58E+01	n.d	n.d
**Spleen**	n.d	n.d	n.d	4.72E+01	7.24E+01	3.52E+01

The virus titres were determined by standard plaque assays on CV-1 cells using aliquots of the homogenized organs and were displayed as mean pfu/per gram of organ or tissue. For each organ, two aliquots of 0.1 ml were measured in triplicates.

n. d.: not detected (detection LIMIT *>*10 pfu/organ).

**Figure 7:**
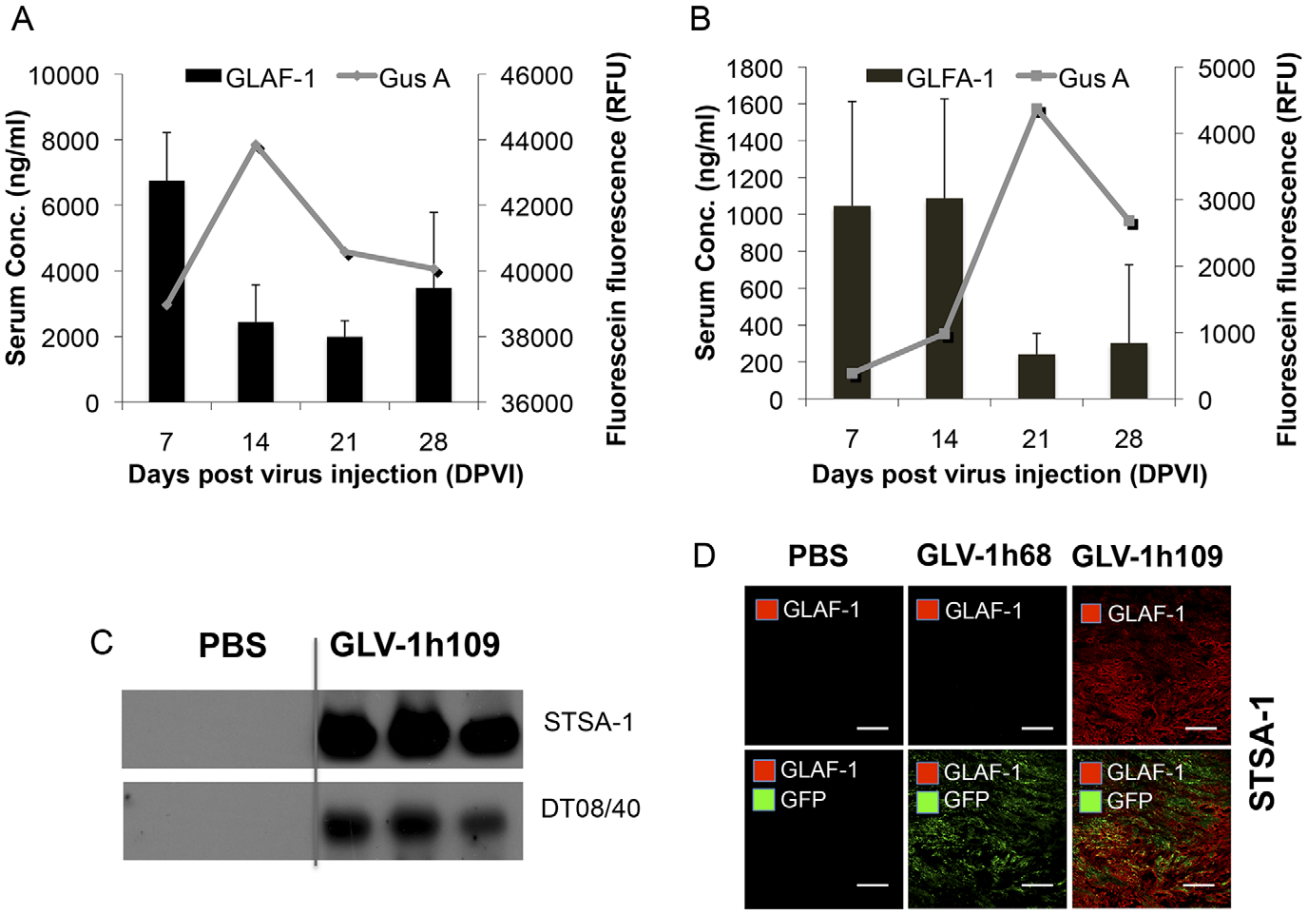
Presence and persistence of the scAb GLAF-1 and GusA in serum (A, B) and tumors (C, D) of GLV-1h109-injected xenograft mice at different time points. A, B: Blood samples were collected at day 7, 14, 21 and 28 from (**A**) STSA-1 and (**B**) DT08/40 tumor bearing mice (n = 6). Expression of GLAF-1 in sera was quantitatively determined using ELISA. GLAF-1 values shown (bars) are mean + SD. GusA activity (represented by lines) was measured by detecting the activation of the fluorogenic compound FDGlcU. **C:** The presence of GLAF-1 in tumor tissue monitored by Western Blot. STSA-1 and DT08/40 tumor-bearing mice injected with GLV-1h109 were sacrificed on day 35 and day 49, respectively. Tumors were collected, and protein fractions from tumor lysate were separated by SDS/PAGE. Western blot analysis was performed using an anti-DDDK antibody. **D:** Localization of GLAF-1 protein in virus-infected STSA-1 tumor areas. Overlays represented the virus infection GFP fluorescence (green)/presence of GLAF-1 (red). Scale bars, 500 µm. (200× magnification).

### Viral Replication

For the viral replication assay, cells grown in 24-well plates were infected with either GLV-1h68 or GLV-1h109 at an MOI of 0.1. After one hour of incubation at 37°C with gentle agitation every 20 min, the infection medium was removed and replaced by a fresh growth medium. After 1, 12, 24, 48, 72 and 96 hours, the cells and supernatants were harvested. Following three freeze-thaw cycles, serial dilutions of the supernatants and lysates were titered by standard plaque assays on CV-1 cells. All samples were measured in triplicate.

**Figure 8:**
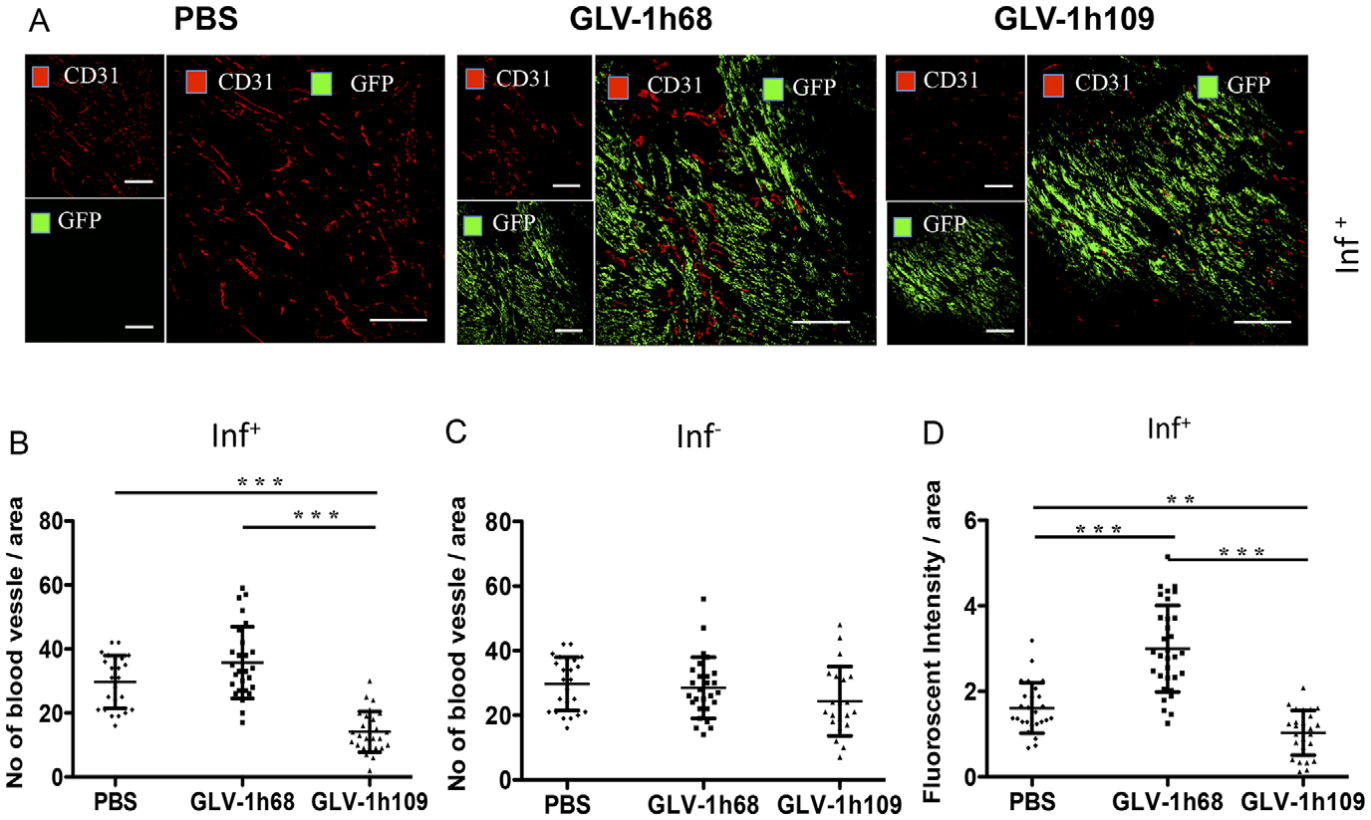
Determination of vascular density using CD31 immunohistochemistry in virus-treated (GLV-1h68, GLV-1h109) and non-treated (PBS) tumors at 7 dpvi. A–C: Blood vessel density in virus-infected (A, B; inf^+^) and virus non-infected (C; inf^-^) STSA-1 tumor areas. The vascular density was measured in CD31-labeled tumor cross-sections (n = 3 mice per group, 18 images per mice) and presented as mean values +/- SD. (***P<0.001, **P<0.01, Student’s t-test). D: Fluorescence intensity of the CD31 signal in virus-infected (inf^+^) STSA-1 tumor areas. The fluorescence intensity of the CD31-labelling represented the average brightness of all vessel-related pixels (VRP) and determined as described [3], [16]. The fluorescence signal is a rate of CD31 expression in the blood vessels and was measured in 18 images of each tumor (n = 3 mice per group). Shown are the mean values +/-SD. (***P<0.001, **P<0.01, Student’s t-test).

### Western Blot Analysis

For detection of proteins, supernatants and infected cells were harvested and resuspended in SDS sample buffer at 24 and 48 hours post virus infection (hpvi). Samples were separated by 10% SDS-Polyacrylamide gel electrophoresis and subsequently transferred onto a nitrocellulose membrane (Whatman GmbH, Dassel, Germany). After blocking in 5% skim milk in PBS, the membrane was incubated with rabbit anti-DDDDK antibody (ab21536, Abcam, Cambridge, UK) for detection of scAb GLAF-1, anti-beta-glucuronidase rabbit polyclonal antibody (G5420, Sigma-Aldrich, Schnelldorf, Germany) or anti-beta-actin mouse monoclonal antibody (ab6276, Abcam, Cambridge, UK). The first antibodies were detected using horseradish peroxidase-conjugated anti-rabbit (ab6721, Abcam, Cambridge, UK) or anti-mouse (ab6728, Abcam, Cambridge, UK) secondary antibodies, followed by enhanced chemiluminescence detection.

**Figure 9:**
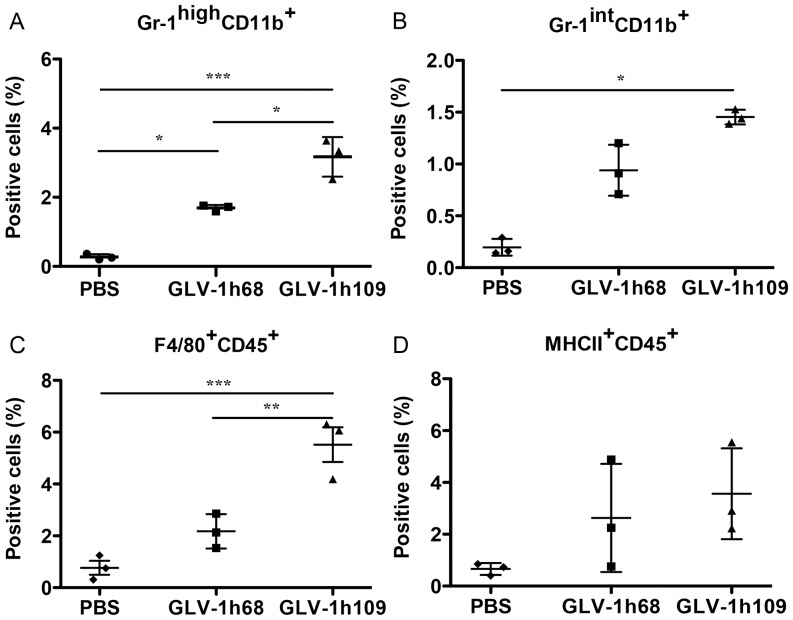
Presence of immune cells in tumors of STSA-1 xenografted mice 7 days after GLV-1h68-, GLV-1h109- or PBS-treatments. Percentage of (A) Gr-1^high^CD11b^+^ (MDSCs, granulocytes), (B) Gr-1^int^CD11b^+^ (MDSCs, monocytes), (C) F4/80^+^CD45^+^ (macrophages) or (D) MHCII^+^CD45^+^ (mainly B cells, macrophages and dendritic cells) cells in tumors of STSA-1 xenografted mice. Experiments were done twice with at least 3 mice per group. The data are presented as mean values +/- SD. The statistical significance was analyzed using one-way ANOVA followed Bonferronìs multiple comparison test (***P<0.001, **P<0.01, *P<0.05). The Anti-Gr-1 mAb (RB6-8C5) has long been used to stain MDSCs and allows the distinction of at least two subsets of granulocytes (Gr-1^high^CD11b^+^) and monocytic cells (Gr-1^int^CD11b^+^).

### Vaccinia Virus-mediated Therapy of STSA-1 and DT08/40 Xenografts

Tumors were generated by implanting either 1×10^6^ canine soft tissue sarcoma STSA-1 cells or 5×10^6^ canine prostate DT08/40 cells subcutaneously into the right hind leg of 6- to 8-week-old female nude mice [Hsd: Athymic Nude-*Foxn1* ^nu^; Harlan, Holland]. Tumor growth was monitored weekly in two dimensions using a digital caliper. Tumor volume was calculated as [(length × width^2^)/2]. When tumor volume reached approximately 600–1000 mm^3^ (STSA-1) and 200–400 mm^3^ (DT08/40), groups of mice (n = 6) were injected either with 5×10^6^ pfu of GLV-1h109 virus or PBS (control) into the tail vein. The significance of the results was calculated by Student’s t-test. Results are displayed as means +/- standard deviation (SD). P values of <0.05 were considered significant.

**Figure 10:**
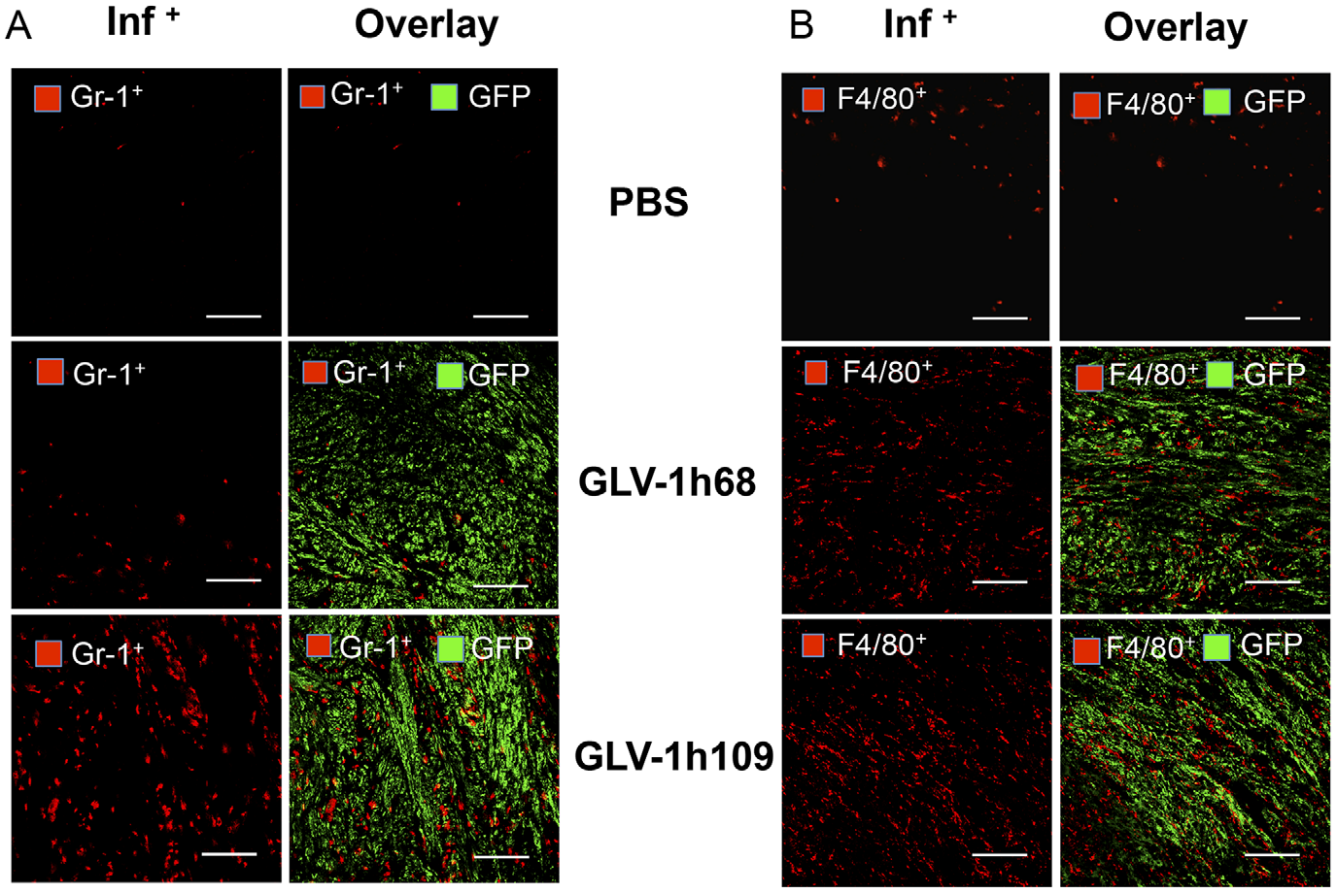
Immunohistochemical staining of infected and uninfected STSA-1 xenograft tumors at 7 dpvi for MDSCs (A) or macrophages (B). Tumor-bearing mice were either infected with GLV-1h109 or GLV-1h68 or mock treated (PBS). Cryosections (10 µm-thick) of tumors were labeled with either anti- Gr-1 (Ly-6G) antibody (**A**) for MDSCs (granulocytes) or anti- F4/80^+^ antibody (**B**) for macrophages; both red. Virus infection and/or phagocytosis was indicated by GFP fluorescence (green). Overlays represented inf^+^(Gr-1^+^)/GFP. Scale bars, 500 µm. (200× magnification).

Mice were monitored for change in body weight and signs of toxicity. Blood samples for protein expression analysis (see below) were collected at time points 7, 14, 21 and 28 days post virus injection under anesthesia by a heparinised capillary pipet (No. 554/20, Assistent, Sondheim, Germany) via the retro orbital sinus vein.

### Fluorogenic Probes and Detection of Fluorescence Products

The lyophilized fluorogenic probe fluorescein di-beta-D-glucosonide (FDGlcU) (Invitrogen, Karlsruhe, Germany) was dissolved in DMSO (36.5 mM). For analysis of beta glucoronidase in serum, the collected mouse serum was diluted 1∶15 with PBS and 80 µl of each sample were mixed with 2.5 µg FDGlcU. After incubation for 1 h at 37°C, fluorescence was read in Lumox 384-well plates (Sarstedt, Nümbrecht, Germany) using an Infinite 200 Pro Microplate Reader (Tecan, Crailsheim, Germany) and fluorescence intensities are listed as relative fluorescence units as described by Hess et al. [15].

### GLAF-1 ELISA

The expression of the recombinant GLAF-1 proteins in sera was quantitatively determined by ELISA. For the standard curve, 6 two-fold serial dilutions of purified GLAF-1 protein ranging from 625 ng/ml to 19.5 ng/ml were prepared in PBS/2% FBS. Purified GLAF-1 protein required to obtain the standard curve was produced as described earlier [7]. Ninety-six well plates pre-coated with recombinant human VEGF (Sigma) were blocked and incubated with standards or 1∶25 dilutions of sera samples in triplicates. Following 1.5 h incubation at room temperature, the wells were washed with PBS/0.05% Tween and incubated with a rabbit anti-DDDDK antibody (ab21536, Abcam, Cambridge, UK) for 1 h at room temperature. All wells were washed and incubated with a secondary HRP-conjugated anti-rabbit IgG (Jackson Immuno Research). Color was developed using 3,3,5,5′-tetramethylbenzidine (Sigma), and the reaction was stopped with HCl. Absorbance was read in an Infinite 200 Pro Microplate Reader (Tecan, Crailsheim, Germany) at 450 nm.

### Histology and Microscopy

For histological studies, tumors were excised and snap-frozen in liquid nitrogen, followed by fixation in 4% paraformaldehyde/PBS at pH 7.4 for 16 h at 4°C. After dehydration in 10% and 30% sucrose (Carl Roth, Karlsruhe, Germany) specimens were embedded in Tissue-Tek® O.C.T. (Sakura Finetek Europe B.V., Alphen aan den Rijn, Netherlands). Tissue samples were sectioned (10 µm thickness) with the cryostat 2800 Frigocut (Leica Microsystems GmbH, Wetzlar, Germany). Labeling of tissue sections was performed as described in detail elsewhere [16], [17].

Endothelial blood vessel cells were stained with a hamster monoclonal anti-CD31 antibody (Chemicon International, Temecula, USA; MAB1398Z). Anti-Mouse Ly-6G (Gr-1, eBioscience, San Diego, USA; 14-5931-81), anti-Mouse F4/80 (Clone BM8, eBioscience) or rabbit anti-DDDDK antibody (ab21536, Abcam, Cambridge, UK) were used to stain myeloid-derived suppressor cells (MDSCs, mainly granulocytes), macrophages or GLAF-1 protein respectively. Cy3- and Cy5-conjugated secondary antibodies (donkey) were obtained from Jackson ImmunoResearch (Pennsylvania, USA).

The fluorescence-labeled preparations were examined using the Leica TCS SP2 AOBS confocal laser microscope equipped with argon, helium-neon and UV laser and the LCS 2.16 software (1024 × 1024 pixel RGB-color images). Digital images were processed with Photoshop 7.0 (Adobe Systems, Mountain View, CA, USA) and merged to yield overlay images.

### Measurement of Blood Vessel Density and Fluorescence Intensity of the CD31 Signal in the Tumor Tissue

Blood vessel density was measured in digital images (100× magnification) of CD31-labelled 10-µm-thick tumor cross-sections using Leica TCS SP2 AOBS confocal laser microscope. Eighteen images per tumor were analyzed per staining (3 tumors per group, 3 sections of each tumor and 6 images per section). Exposure time for individual images was adjusted to ensure clear visibility of all detectable blood vessels and decorated with 8 equidistant horizontal lines using Photoshop 7.0. All blood vessels crossing these lines were counted to obtain the vessel density per section.

Fluorescence intensity of the CD31-labelling in 10-µm-thick sections of control tumors and infected and non-infected areas of virus-colonized tumors was measured on digital images (100× magnification) of specimens stained for CD31 immunoreactivity. On the fluorescence microscope, the background fluorescence was set to a barely detectable level by adjusting the gain of the CCD camera before all the images were captured with identical settings. RGB-images were converted into 8-bit gray scale images (intensity range 0–255) using Photoshop 7.0. The fluorescence intensity of the CD31-labeling represented the average brightness of all vessel-related pixels and was measured using Image J software http://rsbweb.nih.gov/ij.

### Flow Cytometric (FACS) Analysis

For flow cytometric analysis, three mice from each group were sacrificed by CO_2_ inhalation and the tumors were removed. The tumor tissues were minced and incubated individually in 10,000 CDU/ml Collagenase I (Sigma, Steinheim, Germany) and 5 MU/ml DNase I (Calbiochem, Darmstadt, Germany) for 75 min at 37°C and then passed through a 70-µm nylon mesh filter (BD Biosciences, Erembodegem, Belgium).

To block non-specific staining, single cells were preincubated with 0.5 µg of anti-mouse CD16/32 antibody (clone 93, Biolegend, San Diego, USA) per one million cells for 20 min on ice. After that, the cells were incubated at 4°C for 15 min in PBS with 2% FCS in the presence of appropriate dilutions of labeled monoclonal antibodies: anti-mouse MHCII-PE (Clone M5 114.15.2, eBioscience, San Diego, CA, USA), anti-CD11b-PerCPCy5.5 (Clone M1/70, eBioscience, anti-F4/80-APC (Clone BM8, eBioscience), anti-Gr-1-APC (Ly-6G, Clone RB6-8C5, eBioscience). The Anti-Gr-1 mAb (RB6-8C5) has long been used to stain MDSCs and allows the distinction of at least two subsets of granulocytes (Gr-1^high^CD11b^+^) and monocytic cells (Gr-1^int^CD11b^+^) [18].

Stained cells were subsequently analyzed, using an Accuri C6 Cytometer and FACS analysis software CFlow Version 1.0.227.4 (Accuri Cytometers, Inc. Ann Arbor, MI USA).

## Results

### The GLAF-1 Antibody Specifically Recognizes Canine VEGF

The anti-VEGF scAb GLAF-1 is directed against human and murine VEGF [7]. Since until now there were no data about the affinity of GLAF-1 to canine (ca) VEGF, we tested the ability of purified GLAF-1 antibody to bind recombinant ca VEGF (R&D System, Minneapolis, MN, USA) by ELISA. The data demonstrated that this antibody is functional and recognized both ca VEGF and hu VEGF with equal efficiency (**Fig. 1**).

### STSA-1 and DT08/40 Canine Cancer Cells Express VEGF Under Cell Culture Conditions

VEGF, or specially VEGFA is a potent mediator of both angiogenesis and vasculogenesis in dogs and has been proposed as a prognostic indicator in several types of canine cancer [19]–[21]. Therefore we first analyzed the VEGF expression of the two tested canine cancer cell lines under cell culture conditions (**Fig. 2**). Canine VEGF concentrations were determined using a Quantikine ELISA kit (R&D Systems, Minneapolis, MN, USA) developed for detection of canine VEGF, in accordance with the manufacturer’s directions. Concentration of VEGF in supernatant was represented as pg/10^6^ cells. VEGF levels in the supernatant of STSA-1 cells were 1556.92±103.88 pg/10^6^ cells (24 hours) and 2962.19±465.84 pg/10^6^ cells (48 hours), while that of DT08/40 cells were 170.85±6.84 pg/10^6^ cells (24 hours) and 183.30±28.68 pg/10^6^ cells (48 hours). The results revealed that STSA-1 cells produced about 9- to 16-fold more ca VEGF compared to the DT08/40 cells at these two different time points.

### Oncolytic Vaccinia Virus GLV-1h109 Virus Efficiently Kills Canine Soft Tissue Sarcoma (STSA-1) and Prostate Carcinoma (DT08/40) Cells

STSA-1 cells were seeded three days prior to infection in 24-well plates and were then infected with either GLV-1h109 or GLV-1h68 (positive control) at MOIs of 1.0 and 0.1, respectively. Cell viability was analyzed at 24, 48, 72 and 96 hours post-virus-infection (hpvi) by XTT-assays (**Fig. 3A**). Ninety-six hours after GLV-1h109-infection at MOIs of 0.1 and 1.0, only 17.8% and 17.5% STSA-1 cells survived the treatment, respectively. At the same time point and MOIs, we found 18.60% and 18.2% viable STSA-1 cells after GLV-1h68 infection.

The oncolytic potentials of GLV-1h109 and GLV-1h68 were additionally tested in DT08/40 cell culture (**Fig. 3B**). In these experimental settings the GLV-1h109 and GLV-1h68 virus infections led to a similar oncolytic efficacy at 96 hpvi and at MOIs of 0.1 and 1.0.

The results demonstrated that the GLV-1h109 is oncolytic to both canine cancer cell lines, however the rate of oncolysis was faster in STSA-1 cells than DT08/40 cells.

### GLV-1h109 Efficiently Replicates in STSA-1 and DT08/40 Tumor Cells

One of the factors that regulate the oncolytic potential of OVs is their ability to infect and/or efficiently replicate in cancer cells. To determine whether variation in the rate of oncolysis was due to differences in the rate of replication, STSA-1 and DT08/40 cells were infected with either GLV-1h109 or GLV-1h68 at a MOI of 0.1. Standard plaque assay was performed for all samples to determine the viral titers at different time points during the course of infection (**Fig. 4**). The maximum viral titers (total) were observed at 48 hours post virus infection (hpvi) in STSA-1 cells for both GLV-1h68 (2.98×10^6^ pfu/ml) and GLV-1h109 (3.01×10^6^ pfu/ml) (**Fig. 4A**). In addition, GLV-1h68 and GLV-1h109 viruses can also efficiently infect and replicate in DT08/40 cells with a maximum yield of 1.23×10^6^ pfu/ml and 1.56×10^6^ pfu/ml at 72 hpvi respectively (**Fig. 4B**). The highest titer of GLV-1h109 in STSA-1 cells at 48 hrs was nearly twice than highest titer of GLV-1h109 in DT08/40 cells at 72 hrs, indicating that GLV-1h109 replicates better and faster in STSA-1 cells (***P = 0.00004; Student’s t-test).

However, the replication efficiency of GLAF-1 expressing GLV-1h109 strain was similar to that of the parental GLV-1h68 virus in both the canine cancer cell lines.

### GLV-1h109-infected STSA-1 and DT08/40 Cells Express scAb GLAF-1 and Beta Glucuronidase (GusA)

The efficiency of viral expression was also monitored by detecting anti-VEGF scAb GLAF-1 and beta glucuronidase (GusA) proteins in GLV-1h109 infected STSA-1 or DT08/40 cells. For this purpose, 10^6^ ****STSA-1 or DT08/40 cells were infected either with GLV-1h109 or GLV-1h68 (control) at an MOI of 1.0 in 6 well plates. Supernatants and lysates were harvested and analyzed in Western Blot using anti-GLAF-1, anti-GusA or anti-ß-actin antibodies. The ß-actin was used as a loading control and GusA was chosen as an additional protein for monitoring of the viral-dependent protein expression.

The results for virus-infected STSA-1 cells are shown in **Figure 5**. GLAF-1 protein of expected size (30 kDa) was detected in both lysates and supernatants of GLV-1h109-infected STSA-1 cells (**Fig. 5B**). Similar expression of GLAF-1 protein was detected in GLV-1h109 infected DT08/40 (**Figure S1**). No protein of similar size was detected in GLV-1h68 infected or uninfected cells of both cancer types. This is evidence that the GLAF-1 protein was successfully expressed and secreted in both canine cancer cell lines.

### Systemic Administration of GLV-1h109 Virus Significantly Regresses Growth of STSA-1 and DT08/40 Derived Tumors in Nude Mice

Female nude mice [Hsd: Athymic Nude-*Foxn1* ^nu^; Harlan, Holland] (n = 6/group) at an age of 6–8 weeks were implanted with 1×10^6^ STSA-1 cells. Four weeks post implantation, all mice developed tumors with volumes of 600 to 1000 mm^3^. Animals were separated into two groups (n = 6) and were injected with a single dose of GLV-1h109 (5×10^6^ pfu) or PBS (100µl) into the tail vein intravenously (i.v.). As shown in **Fig. 6A** the virus treatment led to a significant tumor regression of all GLV-1h109-virus-treated mice. In contrast, due to excessive tumor burden (>3000 mm^3^), all animals of the PBS control group were euthanized after 14 dpvi.

The therapeutic effect of GLV-1h109 was also evaluated on the progression of canine prostate carcinoma DT08/40 tumors in nude mice by measuring the tumor volume at various time points. The tumors were generated by implanting 5×10^6^ canine prostate carcinoma cells DT08/40 subcutaneously into the right hind leg of 6- to 8-week-old nude mice [Hsd:Athymic Nude-*Foxn1* ^nu^; Harlan, Holland]. Forty-nine days after tumor cell implantation, groups of mice (n = 6/group) were injected (i.v.) either with 5×10^6^ pfu of GLV-1h109 virus or PBS (control). Data demonstrated that a single injection with GLV-1h109 vaccinia virus led to significant inhibition of the tumor growth (*p<0.05) of all virus-treated mice in comparison to the control PBS animals **(** **Fig. 6B**).

Finally, the toxicity of the GLV-1h109 virus was determined by monitoring the relative weight change of mice over time (**Fig. 6C, D**). All GLV-1h109 treated mice showed stable mean weight over the course of studies. There were no signs of virus-mediated toxicity.

### Biodistribution of GLV-1h109 Virus and Presence of scAb GLAF-1 in Tumor-bearing Nude Mice

The GLV-1h109 distribution in STSA-1 and DT08/40 xenografts was analyzed at the last time points after virus treatment. **Table 1** summarizes the virus distribution data in both xenograft models. The highest viral titers were identified in primary tumors of virus-treated mice (**Table 1**). Interestingly, the mean GLV-1h109 titers in primary solid tumors of STSA-1 xenografts at 35 dpvi were about 10^4^ fold higher than that of DT08/40 xenografts at 49 dpvi. In addition, we found presence of plaque forming units in some organs of virus-injected STSA-1 mice, but not in the virus–treated DT08/40 xenografts. However, the number of GLV-1h109 virus particles in the healthy tissues were negligible; e.g. in whole organs: livers (mean weight 1.2 g) about 89 pfu; lungs (mean weight 0.142 g) about 19 pfu and spleens (mean weight 0.2 g) about 10 pfu at 35 dpvi (Table 1, here the pfu were given per gram of organ). In contrast, we found about 10^4^–10^5^ fold more GLV-1h109 pfu in solid tumors at this time point, which clearly shows that GLV-1h109 virus displays an enhanced tumor specific replication.

Using fluorogenic probes activated specifically by virus-mediated glucuronidase (GusA), we have recently shown that the detection of GusA in the serum could be used to evaluate successful tumor colonization and/or transgene expression of oncolytic vaccinia virus in tumor-bearing mice [15]. Therefore, in this study we tested the presence and persistence of scAb GLAF-1 protein in combination with the GusA virus marker (**Fig. 7A, B**).

The maximal yield of scAb GLAF-1 in the serum of GLV-1h109-injected STSA-1 xenografts was about nine-fold higher than corresponding DT08/40 xenografts at 7 dpvi. Interestingly, maximal presence of scAb GLAF-1 protein in serum was a week earlier than the maximal GusA-signal in both the xenograft models (**Fig. 7A, B**). These different kinetics could be due to the fact that GLAF-1 is secreted while GusA is only released to the blood stream via cell lysis. The presence of GLAF-1 in the tumors of GLV-1h109 injected mice was analyzed using Western Blot. The GLAF-1 protein was detected even at the last points of treatment in both xenograft models (**Fig. 7C**). In addition, we clearly detected the GLAF-1 protein in GLV-1h109-treated STSA-1 tumor sections (**Fig. 7D**).

The results demonstrated that analysis of scAb GLAF-1 in the serum could also be used as a pharmacokinetic marker for virus colonization and persistence in GLV-1h109-injected xenograft mice.

### Colonization of GLV-1h109 in STSA-1 Tumor Xenograft Significantly Inhibits Development of Tumor Vasculature

STSA-1 cells express 9–16 times more VEGF than DT08/40 cells as well as expression of scAb GLAF-1 was higher in STSA-1 xenografts than DT08/40 xenografts. In addition, an anti-VEGF strategy was successfully evaluated in dogs with canine soft tissue sarcomas [12]. Considering all these factors, the effects of GLV-1h109 on tumor vasculature and tumor microenvironment were tested in STSA-1 xenograft model only.

To test a possible anti-VEGF effect of the GLAF-1 antibody on tumor angiogenesis and vasculogenesis, we analyzed the CD31-positive vascular network in tissue sections of GLV-1h109, GLV-1h68 and PBS-treated STSA-1 tumors by fluorescence microscopy. For this purpose, CD31-labelled cross sections of tumors from PBS-, GLV-1h68- and GLV-1h109- treated mice were used for determination of the vascular density at the day 7 after treatment (**Fig. 8**). The data revealed that the vascular density of GLV-1h109-infected tumors was significantly decreased in comparison to that of GLV-1h68- and PBS -injected control tumors (GLV-1h109 vs. GLV-1h68 ***P = 0.0000672; GLV-1h109 vs. PBS ***P = 0.000255) (**Fig. 8A, B**). Interestingly, a significant reduction of the vascular density was observed in GFP positive areas (**Fig. 8AB;** **inf^+^**), but not in the corresponding GFP negative areas of tumor sections (**Fig. 8C** **; inf^-^**), indicating that the reduction in vascular density is mediated by virus infection. The vascular density between infected (**inf^+^**) areas of GLV-1h109 tumor was also significantly lower than non-infected (**inf_._^-^**) areas (**inf.^+^** GLV-1h109 (**Fig. 8B**) vs. **inf^-^** GLV-1h109 (**Fig. 8C**); ***P = 0.000534). In addition, fluorescence intensity of the CD31 signal was measured in immunohistochemically stained (**inf^+^**)-sections of STSA-1 tumors (**Fig. 8D**). The results revealed that the fluorescence intensity (vessel-related pixels) of GLV-1h109 virus-infected tumors was significantly decreased in comparison to GLV-1h68 or PBS-injected control tumors (GLV-1h109 vs. PBS **P = 0.0051; GLV-1h109 vs. GLV-1h68 ***P = 0.00001). This means that only the GLV-1h68 virus colonization led to an up-regulation of CD31 protein. However, there was significant decrease in fluorescence intensity of GLV-1h109-infected tumors, which might be due to the reduction in the vascular density after treatment with this virus.

The results demonstrated that the virus colonization in combination with the scAb GLAF-1 led to local inhibition of the blood vessel development in the GLV-1h109 virus-infected tumor tissue only.

### GLV-1h109 Colonization Induces Massive Infiltration of Innate Immune Cells in STSA-1 Xenografts

To investigate potential roles of innate immune cells in the anti-tumor mechanism, we analyzed the effect of virus infection on host immune cells in tumors of STSA1-tumor-bearing mice. Single cell suspensions prepared from STSA-1 tumors, resected 7 days after the treatment were analyzed by flow cytometry for the presence of host immune cells (**Fig. 9**). The presence of various leukocytes was assessed using cellular antigen-specific markers. We used CD45 (leukocyte common antigen), Gr-1 antigen (Ly6C/Ly6G) of MDSCs, CD11b (Mac-1, mainly myeloid cells), F4/80 (macrophages) and MHC II (B cells, monocytes, macrophages and dendritic cells) to visualize the respective cell types in STSA-1 tumors. The tumor-derived Gr-1^+^CD11b^+^ cells consisted of 2 major subfractions based on differential Gr-1 expression, high (Gr-1^high)^ and intermediate (Gr-1^int^). It is known that a Gr-1^high^, mainly composed of immature and mature granulocytes, and a Gr-1^int^, comprising monocytes and other immature myeloid cells [18]. In these experimental settings we found a significant increase of Gr-1^high^ CD11b^+^, Gr-1^int^ CD11b^+^ and F4/80^+^CD45^+^ cells in the GLV-1h109 infected tumors compared with GLV-1h68- or PBS-injected tumors (**Fig. 9A, B, C**). No significant differences were determined in the percentage of MHCII^+^CD45^+^ cells within GLV-1h109 and GLV-1h68 treated tumors (**Fig. 9D**).

An additional immunohistochemical examination confirmed the increased accumulation of MDSCs and macrophages in GLV-1h109-infected tumors as compared to PBS or GLV-1h68-infected tumors (**Fig. 10**). Interestingly, the MDSCs (mainly Gr-1^high^ granulocytes) cells were mostly co-localized with virus in infected tumor regions (**Fig. 10A**), whereas the macrophages were diffusely distributed throughout the tumor (**Fig. 10B**).

We used the Gr-1^high^ CD11b^+^ cells as markers for monitoring viral infection on a systemic level. For this purpose, a parallel flow cytometric analysis of Gr-1^high^ CD11b^+^ on peripheral blood was performed. There was no significant difference between the number of the Gr-1^high^ CD11b^+^cells in peripheral blood of the viruses or PBS-infected STSA-1 xenografted mice (**Figure S2**). These data suggest that the changes in granulocytic MDSCs were not systemic, but rather due to a change in recruitment and/or persistence within the tumors.

Finally, we also analyzed the direct virus interaction with cells of the host immune system at 7 dpvi. At this time point, 0.11% and 0.37% of Gr-1^high^ CD11b^+^ (MDSCs, granulocytes), 0.06% and 0.15% of Gr-1^int^CD11b^+^ (MDSCs, monocytes) and 0.24% and 0.81% of F4/80^+^CD45^+^ (macrophages) were GFP-positive in GLV-1h68 and GLV-1h109-infected tumors, respectively (**Table 2**). This indicates that either these immune cells were infected with vaccinia virus or they had phagocytized virus-infected tumor cells. We found also that GLV-1h109 more efficiently infects both tumor and host immune cells, compared to GLV-1h68 (**Table 2**).

**Table 2:** Percentage of GFP positive cells in virus–treated STSA-1 tumors.

Cell Markers	Virus GLV-1h68	Virus GLV-1h109	P-value GVL-1h68 vs.GVL-1h109	Positive cells
**GFP^+^**	8.67% ±4.5%	14.27% ±1.8%	*(P = 0.039)	total GFP-positive cells
**GFP^+^/** **Gr-1^high^CD11b^+^**	0.11% ±0.073%	0.37% ±0.051%	**(P = 0.006)	GFP-positive tumor associated MDSCs (granulocytes)
**GFP^+^/** **Gr-1^int^CD11b^+^**	0.06% ±0.043%	0.15% ±0.073%	*(P = 0.042)	GFP-positive tumor associated MDSCs (monocytes)
**GFP^+^/F4/80^+^CD45^+^**	0.24% ±0.072%	0.81% ±0.28%	*(P = 0.028)	GFP-positive tumor associated macrophges

Percentage GFP-positive cells in tumors of STSA-1 xenografted mice 7 days after GLV-1h109- or GLV-1h68-treatments. Experiments were done twice with at least 3 mice per group. The data are presented as mean values +/- standard deviations. The statistical significance was analyzed using two-tailed unpaired Student’s test (**P<0.01, *P<0.05).

## Discussion

Several oncolytic viruses including adenovirus strains CAV-1 and CAV-2 [22], canine distemper virus [23] and vaccinia virus strains GLV-1h68 and LIVP1.1.1 [4], [24], [25] have been used for canine cancer therapy in preclinical studies [3], [26]. Our data demonstrated that treatment with oncolytic vaccinia virus GLV-1h109 carrying anti-VEGF scAb GLAF-1 regresses the growth of canine prostate carcinoma and canine soft tissue sarcoma xenografts by oncolysis, inhibition of tumor angiogenesis and recruitment of innate immune cells into tumor tissue.

In the current study, we investigated for the first time the oncolytic vaccinia virus strain GLV-1h109 expressing an anti-VEGF single-chain antibody GLAF-1 as a possible therapeutic agent against canine soft tissue sarcoma and canine prostate carcinoma. GLV-1h109 was able to effectively infect, replicate in and lyse these canine cancer cells in culture. The virus infection led to expression of anti-VEGF scAb GLAF-1 protein in both cell lines (**Fig. 5**). Part of the produced scAb GLAF-1 was specifically secreted into the supernatant of the virus-infected cells (**Fig. 5** **,** **Figure S1**). The secretion of scAb GLAF-1 by the infected cell might lead to its quick delivery to surrounding tumor tissue and further binding to VEGF (**Fig. 7C**). In addition, tumor-specific virus infection led to a continued presence of scAb GLAF-1 in peripheral blood (**Fig. 7A and B**). Data clearly demonstrated that scAb GLAF-1 could be useful as a pharmacokinetic marker for virus colonization and persistence in GLV-1h109-injected xenograft mice (**Fig. 7**). These findings have also very well demonstrated the application of our virus-based system for efficient expression and distribution of recombinant antibodies in the tumorigenic host. Recently we have reported that virus-encoded scAb GLAF-1 led to an enhanced therapeutic effect in different human tumor xenograft models, compared with oncolytic viral therapy with the prototype strain GLV-1h68 alone [7]. We have postulated that this enhancement may be caused by the continuous production of the anti-VEGF scAb in colonized tumors. This possible VEGF blockade and the proximate anti-angiogenesis effects would enhance therapeutic efficacy. In order to test this assumption, we analysed the virus-mediated oncolytic and immunological effects of GLV-1h109 in xenograft mice bearing STSA-1 tumors. The data have revealed that GLV-1h109 achieved a significant inhibition of tumor growth and damage of tumor tissues in both tested canine xenografts models. One of the most important purposes of the study was to determine whether the virus-encoded scAb GLAF-1 could elicit therapeutic anti-VEGF responses. We selected STSA-1 xenografted mice to study the effects of scAb GLAF-1 on tumor angiogenesis, because the canine soft tissue sarcoma STSA-1 cells have shown a high level of VEGF expression (**Fig. 2**). In addition, we observed a nine-fold higher expression of scAb GLAF-1 in the serum of GLV-1h109-injected STSA-1 xenografted mice than corresponding DT08/40 xenograft-bearing animals at 7 dpvi (**Fig. 7A and 7B**). However, higher serum GLAF-1 concentration in STSA-1 xenografts might be due to the enhanced replication efficacy of GLV-1h109 in STSA-1 cells (**Fig. 4**).

A CD31 immunohistological staining of STSA-1 tumor sections revealed a significant decrease in the number of blood vessels of GLV-1h109 infected tumors, when compared to GLV-1h68- and PBS-injected control tumors at 7 dpvi (**Fig. 8**). The drastic reduction of the vascular density of tumors might be due to the presence of the scAb GLAF-1 in tumor tissue and blood of the GLV-1h109-treated STSA-1 mice. Interestingly, the significant reduction in vascular density was observed in virus-infected areas only. It is considerable that tumor microenvironment characterized by interstitial hypertension interferes with intratumoral spread (diffusion) of therapeutic agents [27]. The increased interstitial hypertension in the tumor tissue is the result of abnormal vasculature. Unlike normal blood vessels, tumor vasculature is structurally and functionally abnormal [28]. This abnormal vasculature in STSA-1 tumor xenograft might inhibit intratumoral spread of GLAF-1 protein, which could be the reason for its localized effect. Moreover, effect of GLAF-1 showing decreased vascular density in STSA-1 tumor xengrafts is well supported by the previous findings which demonstrated that the treatment with GLV-1h68 and another GLAF-1-negative oncolytic vaccinia virus strain LIVP1.1.1, did not affect the blood vessel density of STSA-1 tumors [4]. We have also shown that GLAF-1 could specifically bind to canine VEGF (**Fig. 1**). Cross reactivity of GLAF-1 with VEGFs from other species apart from human and mouse was not tested. It was important to know whether GLAF-1 could bind and inhibit canine VEGF. The GLAF-1-binding to VEGF from both canine and mouse origins is advantageous in our canine xenograft models, as blocking of the two VEGF forms could be important for therapeutic efficacy [29].

To investigate the GLV-1h109 virus interactions with the host immune cells we analyzed the innate immune response in the early phase of virus infection by flow cytometry. Data demonstrated a significantly increased accumulation of host immune cells including granulocytic MDSCs and macrophages in GLV-1h109–infected tumors as compared to PBS or GLV-1h68-infected tumors (**Fig. 9**). On the other hand, a significant reduction of the blood vessel density of GLV-1h109 treated STSA-1 tumors was observed when compared to both controls; GLV-1h68 and PBS (**Fig. 8**). A possible reason for this phenomenon may be that the GLV-1h109-treatment led to “vascular normalization” in tumor tissue, as described by Winker and colleagues [30]. The “changed” vasculature seems to allow an increased intratumoral infiltration of MDSCs and macrophages in the tumor bed. Interestingly, MDSCs and macrophages may be associated with both pro- and anti-tumoral activities (for reviews, see [31]–[34]).

Several recent studies have described that virotherapy with vaccinia viruses induces massive tumoral infiltration of MDSCs resembling neutrophils, that may be exerting antitumor effects in vivo through a number of different mechanisms [35]–[37]. In this context, Breitbach and colleagues have postulated that neutrophils (Gr-1^+^ cells) could mediate antitumor effects by the induction of vascular collapse in tumors [36]. In addition, a recent study has identified a cytotoxic population (N1) of tumor associated neutrophils expressing CD11b^+^Ly6G^+^, capable of killing tumor cells [38]. In the current work, we found evidence of direct interactions of vaccinia virus or virus-infected cells with MDSCs and macrophages in the STSA-1 tumor tissue (Table 2; GFP-positive cells). Others and we have recently reported that these interactions may increase the activation and strength of host antitumor immune responses [4], [37]–[39]. Therefore, we think that the interactions of MDSCs and macrophages with vaccinia virus in the tumor bed may be crucial for the success of the virotherapy.

In conclusion, systemic administration of oncolytic vaccinia virus GLV-1h109 expressing an anti-VEGF single-chain antibody led to significant inhibition of angiogenesis and tumor growth as well as to increased infiltration of innate immune cells in the treated canine tumors.

## Supporting Information

Figure S1 **Expression of GLV-1h109 mediated proteins GLAF-1 and GusA in** **canine soft tissue sarcoma DT08/40 cells.** DT08/40 cells were infected with either GLV-1h109, GLV1h 68 virus at an MOI of 1 or PBS. Protein fractions from cell lysate and culture supernatant were isolated at different time points and separated by SDS/PAGE. Western blot analysis was performed using an anti-DDDDK antibody against scAb GLAF-1 and anti-GusA antibody as described in material and methods.
(TIF)

Figure S2 **Presence of Gr-1^high^ CD11b^+^cells** **(granulocytic MDSCs) in peripheral blood of** **virus-infected and non-infected STSA-1 xenografts at 7 dpvi.** Experiments were done twice with at least 3 mice per group. The data are presented as mean values +/- SD.
(TIF)
