# Supplementary figures and images for: Virotherapy of Canine Tumors with Oncolytic Vaccinia Virus GLV-1h109 Expressing an Anti-VEGF Single-Chain Antibody
Source: PLoS One. 2012 Oct 16;7(10):e47472. doi: 10.1371/journal.pone.0047472 (PMC3473019; doi:10.1371/journal.pone.0047472)

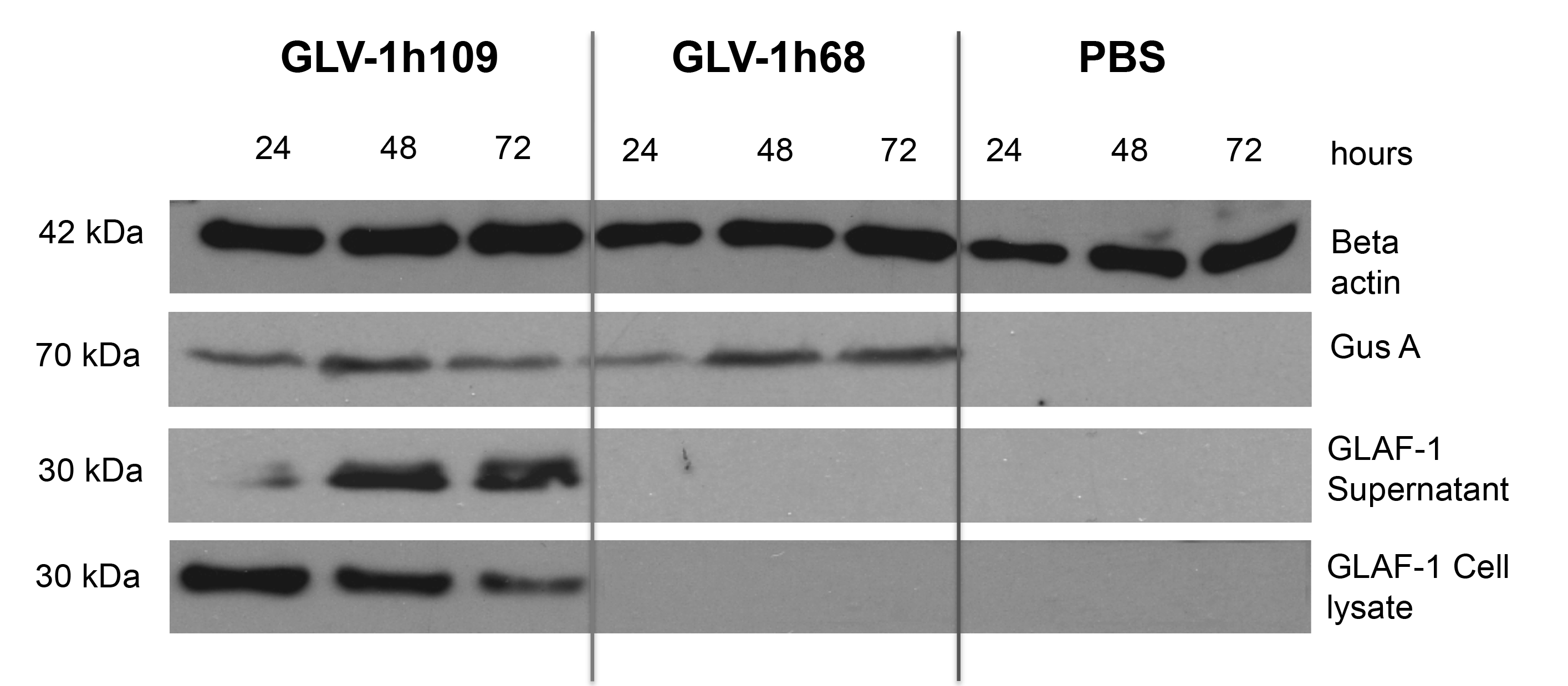

Supplement: Figure S1 — Expression of GLV-1h109 mediated proteins GLAF-1 and GusA in canine soft tissue sarcoma DT08/40 cells. DT08/40 cells were infected with either GLV-1h109, GLV1h 68 virus at an MOI of 1 or PBS. Protein fractions from cell lysate and culture supernatant were isolated at different time points and separated by SDS/PAGE. Western blot analysis was performed using an anti-DDDDK antibody against scAb GLAF-1 and anti-GusA antibody as described in material and methods. (TIF) [file pone.0047472.s001.tif]
